# Artificial Grass as an Alternative Laneway Surface for Dairy Cows Walking to Pasture

**DOI:** 10.3390/ani9110891

**Published:** 2019-11-01

**Authors:** Stephanie Buijs, Gillian Scoley, Deborah McConnell

**Affiliations:** Agriculture Branch, Sustainable Agri-Food Sciences Division, Agri-Food and Biosciences Institute, Hillsborough BT26 6DR, UK; gillian.scoley@afbini.gov.uk (G.S.); deborah.mcconnell@afbini.gov.uk (D.M.)

**Keywords:** gait analysis, walking speed, stride length, preference, floor type, lameness

## Abstract

**Simple Summary:**

Comfortable walking surfaces are of great importance for dairy cow welfare, especially because many dairy cows are affected by lameness, which is associated with painful claws. Cows walk considerable distances to get from their pasture to the milking parlour and back (usually twice a day). Therefore, a more comfortable surface on the laneway between these places will improve their welfare. In this study, we showed that dairy cows walk faster when artificial grass is placed over a standard laneway (consisting of gravel covered with a layer of stone dust) than if this laneway is left bare. This suggests that artificial grass is a more comfortable surface type. In addition, when given the opportunity to choose between the two surface types, the cows more often chose the artificial grass than the standard surface, indicating that they preferred the artificial grass. Lame cows chose the artificial grass more often than sound cows, which is in line with the suggestion that more comfortable surface types are especially important for lame cows. In conclusion, artificial grass provides dairy cows with a more comfortable walking surface, which is likely to benefit their welfare.

**Abstract:**

Softer surfaces can alleviate pressure on the claw during claw–surface contact, which is especially important for cows with painful claws. The benefits of softer barn floors are well known, but as cows often walk long distances twice daily between pasture and parlour, laneway surfaces are also important. In trial 1, we evaluated the gait of 69 cows on a standard (stone dust-over-gravel) laneway and an artificial grass laneway. Greater speed and longer strides were interpreted as indicators of a more suitable surface. Walking speed was greater on artificial grass than on the standard laneway (*p* = 0.001, median artificial grass: 1.46 m/s [interquartile range (IQR): 1.39–1.54], standard 1.40 m/s [IQR: 1.30–1.48]). No significant stride length increase was detected (*p* > 0.10, 158 cm [IQR:151–166] versus 155 cm [IQR:149–164]). In trial 2, we evaluated cow preference by giving 66 pairs of cows four consecutive choices between the standard laneway and artificial grass. Artificial grass was preferred overall (median stretches of artificial grass used out of a maximum of 4: 3 [IQR:2–4], *p* < 0.001). This preference was significantly (*p* = 0.001) stronger in lame cows (median: 3 [IQR:3–4]), than in sound ones (median: 2 [IQR:2–3]). Preference was also affected by the side of the laneway covered with artificial grass. Our results suggest that artificial grass improves the welfare of dairy cows walking to and from pasture, with lame cows benefiting to a greater extent.

## 1. Introduction

Dairy cows’ gait characteristics are affected by their claw health as well as the surface on which they are walking. Claw problems cause lameness, which is one of the most important dairy cow welfare issues [1] and is associated with a decrease in walking speed and stride length [2]. For instance, mildly and moderately lame cows have been shown to walk slower than sound ones (5% and 11% slower, respectively), and moderately lame cows use shorter strides than sound and mildly lame ones (10% and 8% shorter, respectively) [3]. Lameness is commonly associated with painful claws (in 67% of the cases) [4]. Specific painful claw conditions have also been shown to decrease walking speed and stride length (e.g., cows with sole ulcers walked 19% slower using 7% shorter strides [5]). Painful claws can also occur on sound limbs, although this is less common (23% of the cases) and the pain is less severe [4]. Floor type also affects walking speed and stride length, which is at least partially due to alleviation of discomfort by softer (i.e., more compressible), less abrasive floor types [6]. Cows have been estimated to walk 4–6% faster on rubber than on concrete [6,7,8] (although in one study, a higher walking speed was only noted when both floor types were slatted [3]). Stride length has been estimated to be 3–5% longer on rubber than on concrete [3,6,8,9]. In line with the suggestion that softer floor types alleviate some of the pain associated with lameness, cows with more severe signs of lameness showed the greatest increase in stride length when walking on rubber instead of concrete [6], and cows’ mobility score is improved when walking on rubber [7]. It has been suggested that cows reduce their speed when walking on concrete floors to reduce the impact on the hooves when these hit the floor [7]. 

Although increased walking speed and stride length often coincide, this is not necessarily the case. For instance, studies comparing solid rubber to solid concrete reported that stride length was greater on rubber, but speed was higher on concrete [3], or that stride length increased significantly, whereas only a numerical increase occurred for walking speed [9]. In some cases, discrepancies between walking speed and stride length may occur because the tested floor types differ not only in softness and abrasiveness, but also in other characteristics. Slipperiness also affects gait characteristics, which is likely because cows adjust their gait to reduce the chance of slipping and falling [8,10]. Slipperiness is itself affected by the softness and abrasiveness of the floor. Softer floors that claws can sink into have been suggested to be the most effective in reducing slipperiness, and also reduce discomfort due to pressure on the claws [8,9]. Thus, both characteristics would be expected to influence walking speed and stride length in the same direction. However, slipperiness can also be reduced by making a floor more abrasive, which may make claw–surface contact more aversive. When bauxite aggregates were added to an epoxy resin floor, making it both less slippery and more abrasive, stride length was 5% greater, but walking speed was 5% lower. The increased stride length was interpreted as an attempt to minimise contact with the floor [11]. The friction and abrasiveness of a floor is reported to be more strongly correlated to stride length than to walking speed [9].

As the relationship between gait characteristics and how a cow experiences a walking surface is complex, assessing cows’ preferences for different floor types can provide important additional information, allowing a more correct interpretation of changes in gait. Dairy cows are likely to avoid less comfortable surfaces, and are known to prefer rubber over concrete flooring, both when standing and walking [12]. Although this preference would be expected to be stronger in cows with painful claws, the opposite was found when cows’ preferences were evaluated in a group setting. This may have been due to competition between sound cows and lame ones, during which the sound cows would be more successful in claiming a space on the preferred floor type, as lameness reduces dominance rank [12]. Cows showed no significant preference for epoxy resin floors with and without added aggregates (which decreased slipperiness but increased abrasiveness) [13]. This may indicate that although cows will adapt their walking pattern to such floors [11], they can do so in a satisfactory way and therefore feel no need to avoid these.

Previous research on floor types has focused on barn floors. However, cows often walk considerable distances on outdoor laneways whilst travelling between the milking parlour, pasture, and house. Thus, the walking surface on the laneway is likely to affect their welfare. Furthermore, such a surface may improve walking speed, allowing the cow to reach the pasture more quickly and thus increasing the daily time spent on pasture. Artificial grass is used as an alternative laneway surface type on some farms in the United Kingdom. Compared to standard laneways consisting of gravel covered with a stone dust top layer, artificial grass is more compressible. However, as the artificial blades of grass are kept in shape by an infill of sand and small particles of rubber, this material could be perceived as abrasive by the cow. In the current study, we first evaluated cows’ walking speed and stride length on artificial grass laneways and standard laneways. We expected the artificial grass to increase walking speed as it is more compressible. The effect on stride length was more difficult to predict. However, as several studies show an increase in stride length on softer floors, we expected stride length to be greater on the artificial grass. In a second trial, we evaluated lame and sound cows’ preferences for the artificial grass or the standard surface to facilitate the interpretation of the observed differences in gait.

## 2. Materials and Methods 

### 2.1. Forced Trial

Over 200 dairy cows were used for this trial, although only 69 of these animals would be used in the final analysis (see below). The trial was conducted as the cows returned to pasture after their morning milking on three consecutive days in April 2018 and another three consecutive days in May 2019. Cows were assessed after milking because previous research indicates that differences in walking speed and stride length caused by claw problems are more evident at this time than before milking [14]. We assumed that the effects of a softer walking surface would be affected by milking in a similar manner. 

**Figure 1:**
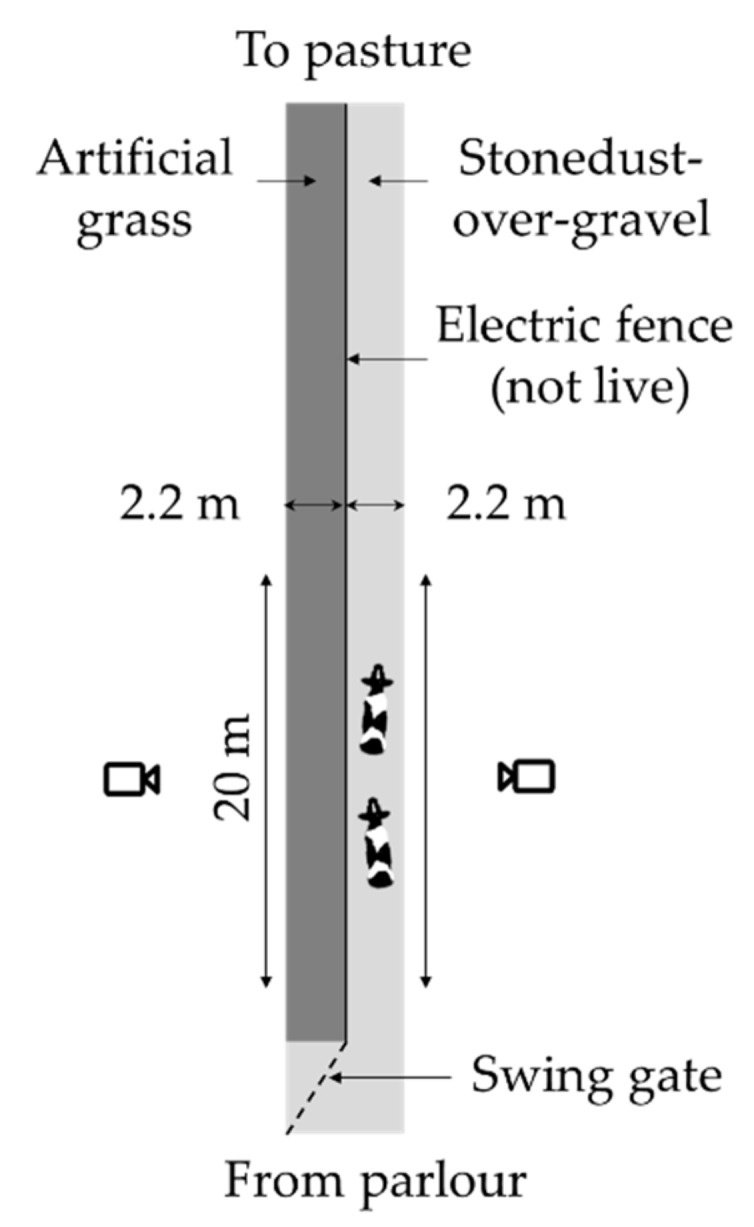
Setup used for the forced trial in which dairy cows’ gait pattern was analysed. The surface types were switched between the two replicates. Figure not to scale.

The experiment was carried out on a 4.4-m wide laneway that was divided into two 2.2-m wide sides (Figure 1). Artificial grass (Astrotracks, Scotland, UK) was placed on top of the stone dust-over-gravel surface on one side of the laneway, whereas the other side was left bare. The artificial grass consisted of an approximately 2-mm plastic base from which plastic “blades” pointed upwards, which were surrounded by an infill of sand and rubber particles. The laneway was split by temporary posts and electric fence wire. After the first three consecutive days of testing, the artificial grass was shifted from one side of the laneway to the other, to avoid confounding between the effects of surface type and side. To habituate the cows to the experimental setup, the artificial grass had been placed on the laneway several weeks ahead of the first set of test days, allowing the cows to use it, but not forcing them to do so. Fencing was put up several days before testing, and all cows passed through the experimental setup at least four times on the days immediately before testing. All cows were forced to use both sides at least once ahead of testing by closing the swing gate. The same habituation procedure was followed ahead of the second set of test days, after the artificial grass had been moved to the other side of the laneway. Between the two sets of test days, all the fencing had been removed, and cows had been able to use both sides of the laneway freely.

Cows were tested in pairs, as testing them individually was expected to lead to problems that would hamper the analysis of their normal walking speed (e.g., stalling, trotting). The pair exited the dairy parlour and proceeded towards the experimental laneway at their own pace. One person followed them at an approximate distance of 5 m until the cows had reached the start of the laneway, and urged the cows on with minimal vocal stimulation and gestures if they stopped before entering the laneway. The next pair was released as soon as the prior pair had passed to the end of the 20-m observation stretch. The swing gate at the start of the laneway blocked access to one of the two sides. The gate was switched after 10 pairs of cows had passed, thus alternately letting 20 cows proceed over the artificial grass and the stone dust-over-gravel side.

Cows were filmed from both sides as they progressed down the laneway, and walking speed and stride length was determined from the videos. To do so, start and finish lines were drawn on the video. These lines were 20 m apart, and markers had been placed at the sides of the laneway to determine their correct location. The time it took the cow at the head of the pair to move from start to finish was recorded to 1/10th of a second (start and finish were considered crossed as the cow moved her first hoof over it). Stride length was determined on the central 10 m of the 20-m trajectory, as the camera’s wide-angle lens resulted in length distortion in the periphery of the video. The distance between the first and last placement of the front hoof within the 10-m trajectory was determined using Kinovea 0.8.15 (www.kinovea.org) and divided by the number of steps to determine stride length. Videos were discarded if a cow stopped, trotted, or cantered on the observation stretch or came within 10 m of the preceding pair (for instance, if this pair was stalling) [15].

For data analysis, paired observations were selected where the same individual walked at the head of the pair over the artificial grass and the stone dust-over-gravel surface within a three-day observation period. Within-pair order was not controlled, nor were pairs specifically selected for a certain surface type on a certain day (but were allowed out on a “first come, first served” basis, with the gate being shifted after 10 pairs). This meant that paired data (i.e., the same cow on both surface types) was available only for a subset of the cows (69 individuals). As cows were tested over three consecutive days in each year, sometimes two observations were available from one individual on one of the surface types within the same year. In this case, the first observation was used. This procedure allowed each cow to be used as her own control and was chosen as we expected substantial individual differences in walking behaviour, regardless of surface type, based on the known effects of cow conformation on stride length [1]. A more systematic ordering of individuals or pairs would have led to delays, causing greater gaps between pairs, which was expected to lead to more trotting to catch up with previous pairs, hampering analysis. Walking speed and stride length were analysed using Wilcoxon signed rank tests in R 3.4.2 [16].

### 2.2. Preference Trial

**Figure 2:**
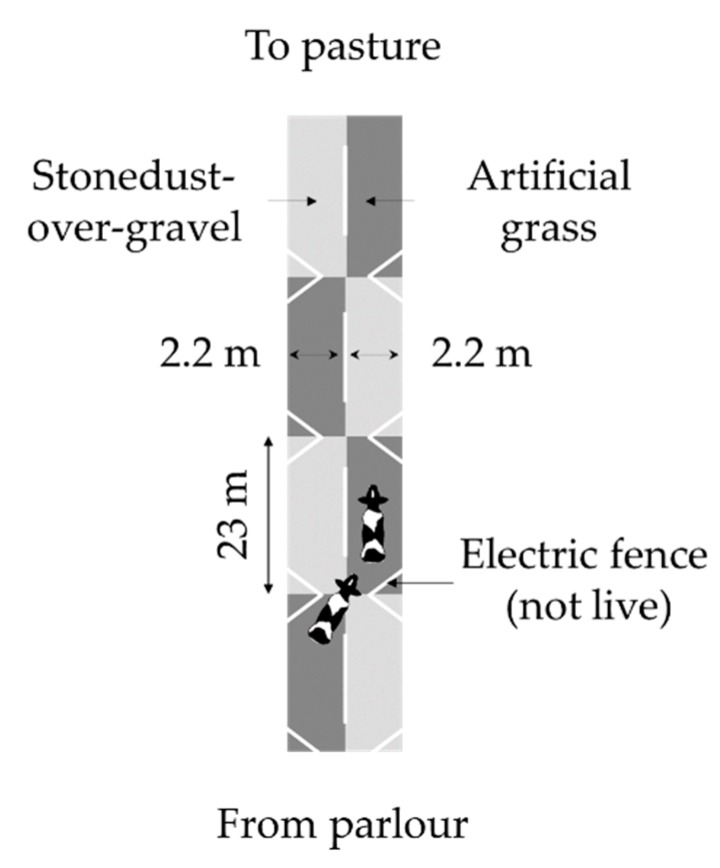
Setup used for the preference trial in which dairy cows’ could choose between surface types. Figure not to scale.

A total of 132 dairy cows was used for the preference trial, although again only 66 of these animals would be used in the final analysis (see below). The trial was conducted on a single day as the animals returned to pasture after their afternoon milking. Upon leaving the parlour, the mobility of all animals was assessed by an experienced assessor using the 1–5 Mason and Leaver scale [17]. To facilitate analysis, these data were dichotomised into sound (mobility score <3) and lame (mobility score ≥3). This meant that slightly lame cows were included in the lame category. This cut-off was chosen as it is known to be scored more consistently than the other categories of the scale [18]. Subsequently, the cows continued on towards the pasture using a stone dust-over-gravel laneway until they were stopped at a spring gate just before the preference test setup. This setup consisted of a dust-over-gravel laneway that was divided into two approximately 2.2-m wide lanes, which were alternatingly covered with approximately 23-m stretches of artificial grass (Astrotracks, Scotland, UK) or left bare (Figure 2). At the point where a stretch of artificial grass started, the two lanes were not separated, and cows were forced to the middle of the laneway using sideways exclusion triangles made of electrical wire. Thus, they had to make a choice at every stretch rather than being able to just walk straight on. Next to the laneway there was a hedgerow on the left side and pastures on the other.

Cows were tested in pairs, as testing them individually was expected to make cows less willing to continue on to pasture at their normal walking speed. In two cases, a third cow managed to join the pair before the gate was closed, and on two occasions, a single cow was tested. Since testing in pairs meant that lane choices were likely not independent, only the data of the lead cow were used (i.e., the cow at the head of the pair, as determined separately for each stretch). Two observers noted the lead cow’s chosen lane from the pasture side of the setup, from a distance of approximately 15 m. On the days and morning prior to the experiment, the cows had been led through the setup several times to habituate them to the setup and allow them to get acquainted with the different surfaces. In addition, they had had several weeks of prior exposure to artificial grass on a different part of the laneway.

Statistical analyses were performed in R 3.4.2 [16]. The number of artificial grass stretches used by each lead cow was summed, and the resulting data were tested against an expected value of 2 (assuming equal use of the two surface types in the absence of a surface preference, with 4 stretches in total) using a one-sample Wilcoxon signed rank test with continuity correction. This was repeated for two separate subsets containing cows with a mobility score <3 (*n* = 43) and ≥3 (*n* = 19), respectively. Four pairs had to be excluded from this last analysis, as a different cow took the lead on the different stretches and the cows in the pair differed in their mobility score. In addition, the number of artificial grass stretches used was compared between the lame and sound subset using a two-sample Wilcoxon rank sum test. As data exploration suggested a preference for the right (pasture) side, analyses were repeated separately for the first and third stretch (where the artificial grass was on the left) and the second and fourth stretch (where the artificial grass was on the right).

## 3. Results

### 3.1. Forced Trial

Data on 31 and 38 lead cows was obtained from the first and second set of test days, respectively. This included 54 Holsteins and 15 Holstein × Jersey crossbreeds, which were 130 days in milk on average (±87 SD) and had an average yield of 33 kg/day (±8 SD). Cows were in their first to eighth lactation (average 2.7 ± 1.8 SD).

**Figure 3:**
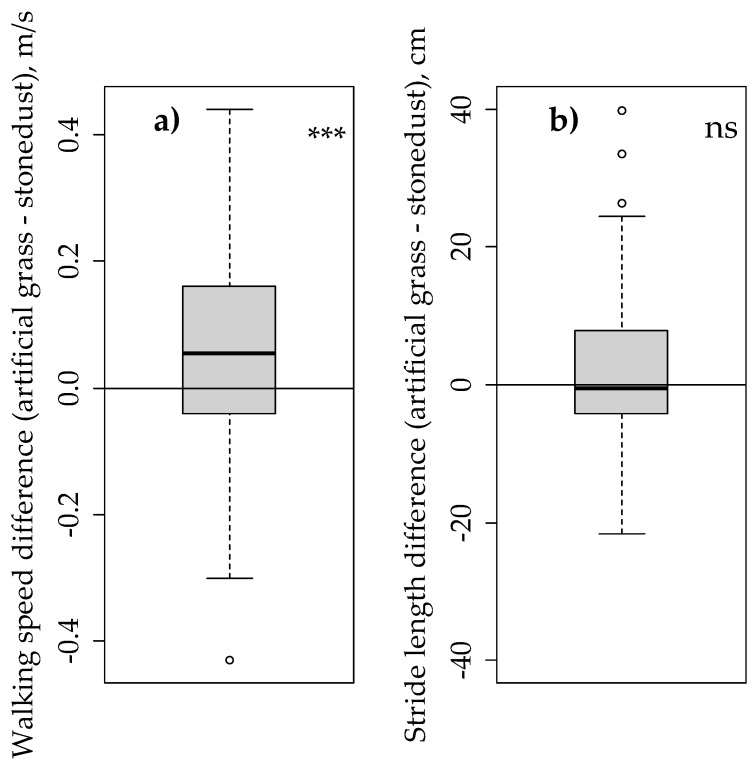
Difference in dairy cow walking behaviour on artificial grass and a standard surface (stone dust-over-gravel), based on 69 pairs of observations. (**a**) Difference in walking speed; (**b**) difference in stride length. *** Significant difference between surface types (*p* = 0.001), ns: No significant difference between surface types (*p* > 0.10).

Cows walked significantly quicker on the artificial grass than on the stone dust laneway (*p* = 0.001, 69 pairs of observations, Figure 3). Median walking speed was 1.46 m/s [interquartile range (IQR): 1.39–1.54] on the artificial grass versus 1.40 m/s [IQR: 1.30–1.48] on the stone dust. No significant difference in stride length was detected (*p* = 0.446, 69 pairs of observations, Figure 3). Median stride length was 158 cm [IQR: 151–166] on artificial grass versus 155 cm [IQR: 149–164] on the stone dust.

### 3.2. Preference Trial

Data on 66 lead cows were used. This included 52 Holsteins and 14 Holstein × Jersey crossbreeds, which were 158 days in milk on average (±108 SD) and had an average yield of 29 kg/day (±7 SD). Cows were in their first to sixth lactation (average 2.8 ± 1.7 SD).

**Figure 4:**
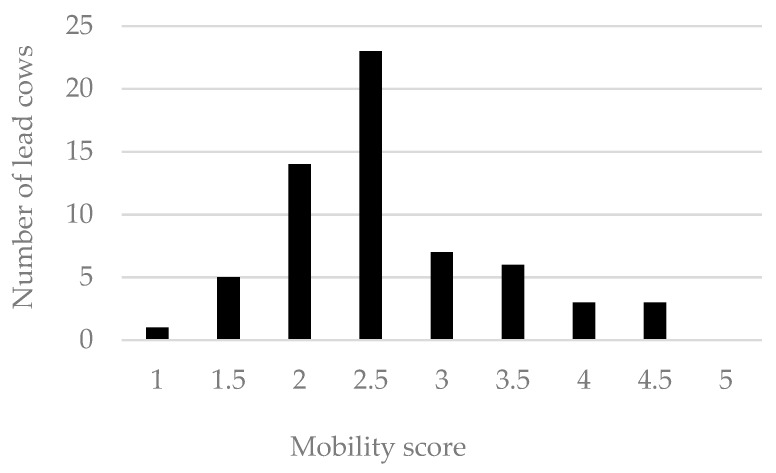
Mobility score of the lead cows of the pairs used in the preference trial. Cows were scored according to Manson and Leaver 1988 [17]. Of the 62 assessed individuals, 43 were classified as sound (mobility <3) and 19 were classified as lame (mobility ≥3). For four additional pairs, the lead cow varied between the stretches, and their mobility score is omitted here.

The distribution of mobility scores of cows leading their pair is shown in Figure 4. 

**Figure 5:**
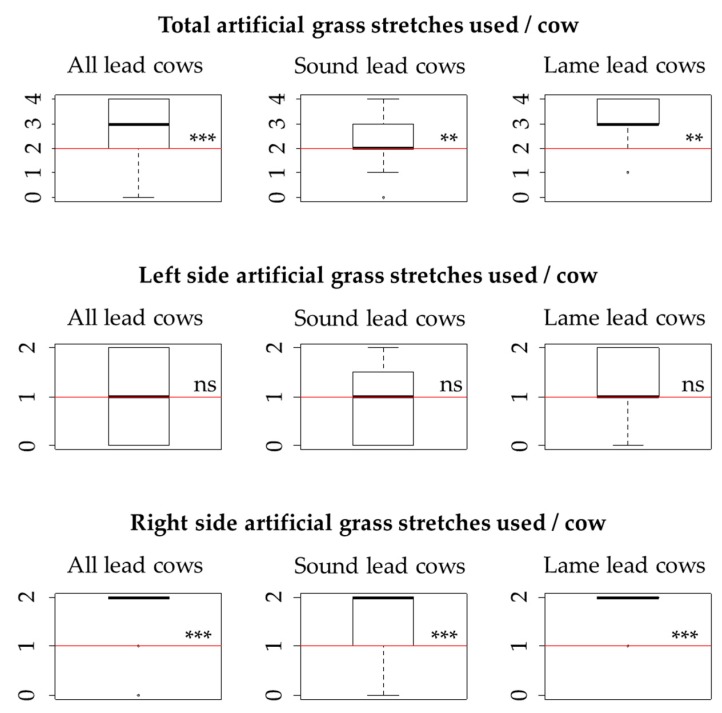
Median number of artificial grass stretches used by lead dairy cows compared to the number expected by chance (expected number displayed as a horizontal line filling the entire width of the frame). The total sample size was 66, including 43 sound lead cows and 19 lame lead cows. The maximum number of artificial grass stretches that could be used was four for the analysis including all stretches, and two for the analyses using either the left-side stretches or the right-side stretches. ** Significantly different from expected values, *p* < 0.001. *** Significantly different from expected values, *p* < 0.01. ns: No significant difference, *p* > 0.10.

Overall, a significant preference for the artificial grass over the stone dust-over-gravel laneway was observed (*p* < 0.001, Figure 5). This was true for sound (mobility score <3) as well as lame (mobility score ≥3) cows. However, the number of artificial grass stretches used by lame cows was significantly greater than the number used by sound cows (lame: 3 [IQR: 3–4], sound: 2 [IQR: 2–3], *p* = 0.013), suggesting a stronger preference for artificial grass in the lame cows. 

Separate analyses showed that the preference for artificial grass was only significant for those stretches where it was placed on the right lane (*p* < 0.001 for all, sound and lame cows, Figure 5), whereas no preference was found when it was placed on the left lane (*p* = 0.882, *p* = 0.343 and *p* = 0.267 for all, sound and lame cows, respectively).

## 4. Discussion

We found that dairy cows walked significantly faster on artificial grass than on a standard, stone dust-over-gravel laneway. The cows also preferred the artificial grass over the standard laneway surface, especially (but not exclusively) when lame. The observed difference in walking speed (4% higher on the artificial grass) is likely too small to result in a substantially increased amount of time spent on pasture. For instance, the decrease in daily walking time for cows that are milked twice daily at 2 km from their pasture would be less than 4 min. However, softer, preferred surface types are known to increase dairy cows’ walking speed by 4–6% [6,7,8,12]. Thus, the 4% increase in walking speed we observed on the artificial grass suggests that this floor type is more comfortable. This interpretation is supported by the finding that our cows showed a significant preference for the artificial grass over the standard laneway (i.e., chose to use the artificial grass more often than the standard surface in the preference trial). This shows that the cows were not walking more quickly on the artificial grass in an attempt to leave an unattractive surface more rapidly. The preference for artificial grass was stronger in lame cows, which was likely a result of painful claws in these animals [4]. Painful claws also occur when sound, although less often and at a lower intensity [4]. This may explain why 44% of our sound cows also chose the artificial grass more often than the standard laneway, although cows may also simply prefer the softer surface even when claw pain is absent. Only a limited number of individuals (12% of sound cows and 5% of the lame cows) chose the standard laneway more often than the artificial grass, and only one (sound) individual avoided the artificial grass altogether. This may indicate an opposite preference in a minority of the cows, suggesting that individuals may differ in how they respond to different surface-type characteristics. Alternatively, these cows may not have been sufficiently habituated to the artificial grass, even though they had all passed it several times in the days before the trial.

In contrast to our expectations, the increased walking speed was not accompanied by a greater stride length. This suggests that the cows increased their walking speed at least partly by moving their legs faster, although leg movement was not quantified separately. Discrepancies in the effect of softer floor types on walking speed and stride length have been noted before [3,9]. One of these studies also showed that slipperiness has a greater impact on stride length than on walking speed [9], and neither of our tested surfaces became noticeably slippery (not even under rainy conditions). This may explain why we observed no difference in stride length. In contrast, the artificial grass did provide a much softer walking surface than the standard laneway, as was clearly noticeable when walking over the material in rubber boots. 

In addition to a preference for the artificial grass, the cows also showed a preference for the right side of the laneway. The reason for this side preference cannot be determined decisively from this experiment, as it was not set up to evaluate this. Cows may have preferred to stay closer to the pastures on the right side of the laneway or may have been avoiding close proximity to the hedgerow on the left. Alternatively, they may have preferred to stay on the right because they had to turn a corner to the right further down on the laneway. However, this corner was located over 50 m after the end of the preference test setup. Furthermore, choosing the right side did not actually lead to a shorter total distance due to the triangular exclosures of the test setup, which forced the cow into the middle of the laneway ahead of each choice. Side preferences occur regularly in preference testing even in completely symmetric setups [19,20], and therefore, our experiment was set up to avoid confounding between the side preference and the surface type preference (i.e., artificial grass stretches were placed alternatingly on the left and the right side). Thus, the side preference does not discredit our findings regarding the preference for artificial grass. It can be concluded that surface type is not the only aspect that influences the average dairy cow’s choice of path, as would be the case if the satisfying the preference was so important that no trade-off would be able to affect it. However, as the cause and the importance of satisfying the side preference remain unclear, the observed trade-off between surface preference and side preference does not provide clear insight into the importance of satisfying the surface-type preference.

In addition to the effects found in this study, long-term benefits may be expected, which are to be evaluated in further research. Prolonged use of softer barn floor types can reduce lameness in dairy cows, heifers, and dairy-origin beef cattle [21,22,23] (although such benefits are not always achieved [24]). Similar benefits could be gained from the prolonged use of artificial grass laneways. Furthermore, a preferred walking surface may make dairy cows more willing to return from the pasture to the parlour in robotic milking systems with pasture access (which require the cow’s voluntary movement between pasture and parlour). Both cow welfare and productivity may benefit from this, as milking frequency is often sub-optimal in such systems [25]. In addition to long-term benefits, there may also be long-term disadvantages, for instance if wear of the hoof is reduced too much by the use of artificial grass leading to overgrown claws. This possibility will require further investigation as well.

Although the current study only focused on short-term effects, both the increased walking speed on artificial grass and the preference for this surface suggest that it is beneficial for dairy cow welfare.
